# “Once you join the streets you will have to do it”: sexual practices of street children and youth in Uasin Gishu County, Kenya

**DOI:** 10.1186/s12978-015-0090-z

**Published:** 2015-11-17

**Authors:** Lonnie Embleton, Juddy Wachira, Allan Kamanda, Violet Naanyu, Susanna Winston, David Ayuku, Paula Braitstein

**Affiliations:** Moi University, College of Health Sciences, School of Medicine, Eldoret, Kenya; Academic Model Providing Access to Healthcare (AMPATH), Eldoret, Kenya; Moi Teaching and Referral Hospital, Eldoret, Kenya; Department of Behavioral Sciences, Moi University, College of Health Sciences, Eldoret, Kenya; Warren Alpert Medical School, Brown University, Providence, RI USA; Rhode Island Hospital/Hasbro Children’s Hospital, Providence, RI USA; Indiana University, Fairbanks School of Public Health, Indianapolis, IN USA; University of Toronto, Dalla Lana School of Public Health, Toronto, ON Canada; Regenstrief Institute, Inc, Indianapolis, IN USA; University of Toronto, Institute of Medical Sciences, Faculty of Medicine, Toronto, ON Canada

**Keywords:** Street children, Street youth, Sex, HIV, Sexual behaviour, Sexual violence, Abuse, Human rights

## Abstract

**Background:**

Adolescents living in HIV endemic settings face unique sexual health risks, and in the context of abject poverty, orphanhood, social marginalization, and discrimination, adolescents may be particularly at-risk of horizontal HIV transmission. Street-connected children and youth are a particularly vulnerable and marginalized population and therefore may be a key population at-risk.

**Methods:**

We sought to describe the sexual behaviours of street-connected children and youth in order to comprehend their sexual practices and elucidate circumstances that put them at increased risk of contracting HIV utilizing qualitative methods from a sample of street-connected children and youth in Eldoret, Kenya. We recruited participants aged 11–24 years who had lived on the street for ≥ 3 months to participate in 25 in-depth interviews and 5 focus group discussions stratified by age and sex.

**Results:**

In total we interviewed 65 street-connected children and youth; 69 % were male with a median age of 18 years (IQR: 14–20.5 years). Participants identified both acceptable and unacceptable sexual acts that occur on the streets between males and females, between males, and between females. We grouped reasons for having sex into four categories based on common themes: pleasure, procreation, transactional, and forced. Transactional sex and multiple concurrent partnerships were frequently described by participants. Rape was endemic to street life for girls.

**Conclusion:**

These findings have important policy and programming implications, specifically for the government of Kenya’s adolescent reproductive health policy, and highlight the need to target out-of-school youth. There is an urgent need for social protection to reduce transactional sex and interventions addressing the epidemic of sexual and gender-based violence.

## Introduction

Approximately 780,000 adolescents aged 15–24 years were newly infected with HIV in 2012, with 72 % of these cases occurring in sub-Saharan Africa, where young people accounted for 39 % of all new HIV infections [1]. Gender inequity is prominent in the number of new HIV infections among young people aged 15–24, with girls being 50 % more likely to acquire HIV than their male peers [1]. Adolescents living in HIV endemic settings face unique sexual health risks, and in the context of abject poverty, orphanhood, social marginalization, and discrimination, adolescents may be particularly at-risk of horizontal HIV transmission [2]. Street connected children and youth are a particularly vulnerable and marginalized population and therefore may be a key population at-risk. The literature indicates the majority of children and youth with street-connections in sub-Saharan African are sexually active [3–7], are subject to sexual violence [8, 9], participate in transactional sex [3, 6, 9, 10], and engage in sex under the influence of substances [11]. Yet, we know little about their sexual behaviours from their perspectives, including what they consider sex, what they call different types of sex, the circumstances surrounding their sexual debut, reasons for engaging in sex, and the outcomes of their sexual partnerships. It is essential to understand these issues in order to know how to talk to these vulnerable young people about sex, sexual risks, and harm reduction, whether for research or care purposes. It is necessary to comprehend how and why street-connected children and youth engage in sexual relationships to elucidate circumstances that put them at increased risk of contracting HIV and to develop adolescent sexual health programs specific to their needs. We therefore sought to describe the sexual behaviours of children and youth connected to the streets utilizing qualitative methods in Eldoret, Kenya.

## Methods

### Human subjects protection

Ethical approval was obtained from the Moi Teaching and Referral Hospital (MTRH) Institutional Research and Ethics Committee (IREC) as well as the Indiana University Institutional Review Board (IRB). All interview sessions were conducted in private rooms. Prior to the interviews, trained research assistants provided street youth with verbal information about the study and sought their participation. Written consent was obtained from willing participants aged 18 years and older. While written assent was required for those aged 11–17 years in addition to written guardian consent from the UG District children’s office. Fingerprints were used for children who were unable to sign or write their names. A child psychologist was present during all interviews sessions for children aged 11–17 years, in order to provide counseling in the event of psychological distress during the sessions. Privacy and confidentially was assured at all times. During the interview sessions participants were requested to talk about their general perceptions and observations about sexual activities in the street community and not about their own personal experiences. In addition, participants were asked not to disclose their full names and/or those of other participants during the sessions. First names of participants were used to facilitate discussions during the FGD sessions.

### Study design and timeframe

This qualitative study was conducted from August 2013 to February 2014. In-depth interviews and focus group discussion (FGDs) were used to collect data.

### Primary study objective

The primary objective of the study was to explore the sexual language and practices of street-connected children and youth in western Kenya. The focus of the present analysis is on sexual practices.

### Study setting

Uasin Gishu (UG) County is one of the 47 counties of Kenya, located in the western part of the country. In 2010, UG County had approximately 894,179 individuals from 202,291 households, of whom 41.5 % were aged 14 years or less. Approximately 51.3 % UG County population live below the Kenyan poverty line. Eldoret town is headquarters of UG county and has a population of 289,389 [12]. It is home to Moi University (including Kenya’s 2nd medical school), Moi Teaching and Referral Hospital (MTRH), and the USAID-AMPATH (Academic Model Providing Access to Healthcare) Partnership [13]. A long-standing partnership between Moi University, Moi Teaching and Referral Hospital, and a consortium of universities led by Indiana University form the AMPATH Consortium. The program is headquartered in Eldoret, Kenya. Through this consortium, there is a large, multi-disciplinary, and well-established research program. The Principal Investigators of the study were from Indiana University and Moi University, respectively, and both live in Eldoret, the capital of the county of Uasin Gishu. The research team has an on-going and well-established relationship with street-connected children and youth in the town and therefore we chose to conduct this study in this geographic region for all of these reasons.

### Study population

We targeted children and youth connected to the streets aged 11–24 years whom had lived on the street for ≥ 3 months. Street-connected children and youth were defined as individuals who either a) were spending both days and nights on the streets, and had limited-to-no parental/guardian contact or b) were spending a portion or majority of their time on the street and had a parent/guardian/caregiver to whom they returned at night or c) a combination of these situations at different times.

### Sampling and recruitment

Study participants (11–24 years) were purposively sampled from three points: 1) A dedicated study clinic for vulnerable children and youth at MTRH; 2) Street venues “bases/barracks” (primary locations in which street children reside); 3) Street youth community-based organizations. Extensive street outreach and study sensitization occurred at these sites to establish rapport and trust with children and youth connected to the streets. A pre-existing relationship between the research team and a number of street-connected children and youth in Eldoret assisted in identification and outreach within these locations. A street outreach worker with extensive experience working with this population in Eldoret was engaged to conduct the outreach and provide information about the study. Prior to recruitment, the street outreach worker visited all of the sites throughout the town known to be bases, as well as to organizations providing services to children in street circumstances to identify all possible locations where street children are found. Convenience sampling was the methodology utilized to recruit participants. During outreach sessions, conducted both individually and with groups in locations where street-connected children and youth reside, the purpose of the study was explained and children were invited to participate voluntarily in the investigation. The street outreach worker would regularly re-visit bases throughout the study to recruit street-connected children and youth interested in participating. Specific sites and areas where street connected girls are known to live and congregate were specifically targeted to recruit this especially hard to reach population.

### Study instruments

Interview guides were developed for in-depth interviews and FGDs and modified for the younger participants (11–13 years) versus older participants (14–24 years) to reflect developmentally appropriate questions regarding sexual activities. An additional interview guide was developed to explore the specific roles among barracks leaders (typically older youth who are seen as authority figures and leaders at a specific barracks and dictate rules of their barracks, sometimes they also take on religious leadership roles). A series of 10–15 open-ended questions previously pre-tested among street-connected children and youth in UG for understandability and completeness guided the individual interviews and FGDs. Interview guide domains and questions included: 1) initiation on the streets (processes involved in being accepted into a street group) (2) types of relationships established on the streets 3) sexual practices (acceptable and unacceptable) and behaviours 4) language used for sexual acts 5) reproductive health (pregnancy, childbearing, abortion, and family planning) 6) sexually transmitted infections and health 7) sexual abuse and rape and 8) roles and responsibilities of leaders on the streets. In addition, a set of structured questions on basic socio-demographic information of age, gender, educational level and occupation were incorporated.

### Procedure

We conducted a total of 25 in-depth interviews and 5 FGDs consisting of 8-12 participants each, stratified by the following age and sex groups: males 11–13 yrs, males 14–17 yrs, males 18–24 years, females 14–17 years and females 18–24 years. Table 1 shows the distribution of interview sessions. We were unable to conduct FGDs or in-depth interviews with females aged 11–13 years due to difficulty in recruitment. Eligible participants were referred to the study clinic at MTRH where all interview sessions were conducted in private rooms. The study clinic provided a neutral, safe, and private location to assist making street-connected children and youth more comfortable attending to discuss sexual and reproductive health practices. Once written consent or assent was obtained, participants were invited to take part in the FGDs. At the end of the FGD session, the study team invited willing participants to further participate in the in-depth interviews. The individual interviews took an average of 40 min while the FGDs took an average of one and half hours. All sessions were audio-recorded and conducted by two of the study investigators in Swahili. For the FGDs, a trained research assistant was present to take notes. Transport reimbursement of 100 KES (~USD = 1.15) was provided after the interview sessions. This amount was considered adequate given the cost participants incurred to get to the clinic without being enough to coerce or influence participation in the study.

**Table 1:** Distribution of interview sessions

Category	# of In-depth interviews participants	# of FGD’s conducted
Barrack leaders	4	0
Male		
11–13 years	1	1
14–17 years	6	1
18–24 years	7	1
Female		
11–13 years	0	0
14–17 years	3	1
18–24 years	4	1
Total	25	5

### Data analyses

Recorded interviews were transcribed and translated to English. The data were then coded and themes related to ideas about sex and sexual practices. Ideas from different interviews were then pooled together and integrated into common themes. Concepts from these themes were generated and used to organize the presentation of the results. For validation, independent coding and identification of themes were conducted by four investigators. The final write up consisted of summaries, interpretations and textual excerpts.

## Results

In total there were 65 children and youth connected to the streets who participated in FGDs and in-depth interviews; 69 % were male with a median age of 18 years (IQR: 14–20.5 years). The majority spent days on the streets and nights in a rented shelter with other street peers (51 %); with very few returning to a parent/guardian at night (24 %). Less than half (43 %) of the participants had attained at least an upper primary school (classes 5–8) level of education. Almost all (81.5 %) participants indicated they were currently sexually active.

### Sexual debut

Commencement of street-involvement, sexual debut, and an inability to remain celibate are intimately linked. Both boys and girls explained that once street-involved they start having sexual relationships, as one girl recounted: “*Any time that one joins the street. They start even at the age of 8. Once you join the streets you will have to do it*” (Female, 21)*.* Street-connected children and youth reported that children as young as 5 years engage in sexual acts.

*“Once you join the street you will automatically start having sex and even if you are 5 years you will start with the young children then when you reach the age of 8 years …some even start at 10…10 aha…some even at 7 or 8 … Aha…where I was yesterday, in kenyaa, I was shocked to see a very young boy having sex. Very young. So it depends. I started at 9 years”* (FGD, Male 18–24).

### Celibacy

Almost unanimously participants indicated that once street-involved it was nearly impossible to remain celibate and that even very young children engage in sexual acts once entering the street. When inquiring regarding celibacy one female participant responded: “*(laughing) but in that street it is not possible. There are no such people on the streets*” (Female, 18).

Age and gender were factors discussed by participants in relation to celibacy. Some participants indicated that perhaps it was possible for boys to remain celibate: “*Yes it is possible and we even have some. There are those boys who usually say they don’t want to have sex until they get married*” (Male, Religious Barracks Leader, 18). However, this occurs very rarely among girls as they are forced to have sex, “*There are those who do not accept, if you are tough. But if you are just a weakling, they will rape you*” (Female, 18). Young age made both boys and girls vulnerable to being forced into sex and therefore unable to remain celibate as one girl explained: “*It depends. If one is a grown up where he/she can decide to do it or not to do it. But if you are still young and you don’t want, you will be beaten and forced to have sex*” (Female, 21).

Sexually transmitted infections may also be a factor for street youth in abstaining from sex as one boy explained: “*They passed through a situation that cannot allow them to participate in sex again. For instance a girl had sex with a boy and contracted a disease that has damaged them completely and they can no longer participate in sex. They fear to engage again in sexual activities*” (Male, 13).

### Sexual acts

Street-connected children and youth engage in various sexual acts, including vaginal intercourse, anal sex between men (men who have sex with men (MSM)), anal sex between men and women, sex between women (women who have sex with women (WSW)), oral sex, and masturbation. Sexual acts may be considered ‘acceptable’ or ‘unacceptable’. Table 2 lists the sexual acts reported by participants, the street vernacular used to describe the act and a quote referring to its context on the street.

**Table 2:** Sexual act terminology used on the streets by street-connected children and youth in Eldoret, Kenya

Sexual Behaviour	Language used on the street	Context on the streets
Heterosexual partners	Mapenzi, Ngono	“The only sex acceptable is for a girl and a man and it is called ‘*mapenzi*’” (Male, 15)
MSM	Mashoga, Mamende, Mameda	“We say ‘*kumendana*’ or ‘*mashoga*’” (Female, 17)
WSW	Semenya, Mang’aa, Shoga wamama, Wamama wa kupigana mafinger, Menda	“‘*Shoga wamama’* If the women are found doing sex among themselves” (Male, 18)
Vaginal Intercourse	Wanapasuana deki, kutombana/kutombwa, kudinyana/kudinya, kupigwa maji/kupigana maji, kupasuana kibwenye, kurwakana, kubinjwa/kubinjana, kutiana, kumangana, kuguzana ololo, kurepiana, kupigwa stiki, -, kuingizwa	“A boy and a girl? They can call it, but mostly they call it ‘*Kutombana*’; that is the name” (Male, 17 years)
Anal Intercourse (MSM), typically forced sex	Kukumenda/kumendana matako/menda, kupigana miti kwa matako, kukonorana/kupigana mabuti, kupigana mzigo ya nyuma, kufira nyuma, kurepiana, kutombwa nyuma	“‘*kumendana*’?…a man forces a boy to give him the buttocks” (Male, FGD 11-13)
Anal Intercourse (heterosexual)	Kumenda, kufirana	*Kumenda* is used when making reference to the penis being inserted into the female anus
		*Kufirana* is used when the male or female fingers are inserted into the female anus
Oral Sex (fellatio)	Kudara, kupapasa, Kunyonya deki	“That goes on because that is their love, so you will find a woman ‘*ananyonya de*ki’” (Barracks Leader)
Oral sex (cunninglingus)	analamba kae’ ‘anamyonya matiti’, kudara	“Or you find a man ‘*analamba kae*’ ‘*anamyonya matiti*’” (Barracks Leader)
Rape (Vaginal)	Kubambwa, kubakwa, mada, muhadhara, kunajisi, kupigwa ngeo, kubinjwa	“We call it ‘*mada*’ because it is like exhausting someone.” (Male, 15)
Male Masturbation	Mashushumen, shushumeni, kujipiga binto, Kujidara	“It happens to those who don’t know how to seduce a girl. They do that, ‘*kujipiga binto*’” (Male, FGD 18-24)
Female Masturbation	Kujipiga doli, wanajirusha dole, mtu anajimanga peke yake	“There are others who do that. ‘*wanajirusha dole*’ Or ‘*mtu anajimanga peke yake*’ Which means a person doing sex alone.” (Female, 17 years)
Transactional sex	Kupeana kuma, Malaya, kujiuza	“‘*kujiuza*’ means you have come from maybe barracks, you have come to paradise or clubs, you have come to look for money because you money is what talks these days..You are broke and so you just go there, so that is what we call ‘*kujiuza*’” (Male, 17)
Gang rape	Kollabo, kombi, kupiga mzigo rende ya watu wengi, kupigwa mzigo na majeshi, raping, kumuangukia	“‘*wanakupiga kombi’* and when you accept you are okay…They do a combination on you…they make a queue for you…base members, very big men” (Female, FGD, 14-17)

#### Acceptable sexual acts

Vaginal intercourse is considered acceptable sex on the streets and the most common sexual act between a man and woman, “*If it is a relationship between a man and a woman, it is normal but if it is between men we don’t agree with it*” (Male, 24). Oral sex between a man and woman is also considered acceptable sex, however, it is not as common, and some participants indicated they did not accept it, “*There are those who do that and it is recognized as sex although with me I cannot accept it*” (Male, 18).

#### Unacceptable sexual acts

While most sexual acts between men and women were generally considered acceptable on the streets, anal sex between men and women was not considered acceptable as one male inferred: “*…on her back side? aiiiiii you can’t do that to a girl!* (Male, 20)”. Yet, another male reported that this occurs and is offered by women, but it is considered unacceptable on streets:

*“It happens. A girl can tell you her vagina has been worked on too much so you can work on her behind…Eeh, she has two holes…you will hear them say they have a shop next to a toilet… (Laughter)…We can tell a girl that she has a shop next to a toilet. The shop is her vagina and the toilet is her buttocks.....no it is not acceptable…eeh…if you get a girl and you reach an agreement, why should you do her from behind?…eeh..”* (FGD, Male, 18–24).

In general, street-connected children and youth explained that sexual relations between two men or two women were unacceptable on the street and not tolerated in the barracks. However, many participants said that sexual acts among MSM occur. Participants also said that while WSW occurs, it is less frequent. There was no mention of oral sex occurring in MSM or WSW partnerships. Sexual encounters between women were uncommon, yet children and youth connected to the streets indicated they have “heard” of women who have done this, and they would be chased away from their barracks if they were caught. As explained by one boy, MSM and WSW sexual relationships are not acceptable on the streets, although they do occur:

*“There are some men who do that (referring to MSM) and it is not acceptable. Even when two girls do sex with each other it is not acceptable. The only sex acceptable is for a girl and a man and it is called “mapenzi” (Swahili for ‘lovers’) Because it is normal”* (Male, 15).

Anal sex that occurs between MSM is not considered acceptable and street-connected children and youth indicated that if you discover a man having sex with another they will be punished:

*“Sex between men is not acceptable because people will get used. So when they are found doing it, they will be beaten. Sex between girls is also not acceptable because the girls have children and the children might end up learning the same character. So if they are also found, they will be beaten”* (Male, 14).

Despite being punishable and unacceptable, one male indicates pedophilic anal sex between older youth/men and young boys commonly occurs, typically by force or through transactional encounters, as reported by one male:

*“Sex between men has been a very common thing although it is the older boys who cheat the small boys and then they sodomise them. It is rare to see two older men doing it between themselves. It is sex but we don’t like it”* (Male, 24).

Masturbation was considered an unacceptable sexual act among children and youth connected to the streets and considered punishable if caught. Boys felt that ejaculating without using the sperm to impregnate a woman was “throwing away” or “killing” as offered by one boy:

*“There are many boys and girls, why should you do that yet God created man to be with a woman…mmh…and you would have thrown away children instead of impregnating a girl, so it is like you have killed and God will judge you…”* (Male, FGD, 18–24).

### Reasons for sex

Street-connected children and youth reported many reasons for engaging in sex. We grouped reasons for having sex into four categories based on common themes: pleasure, procreation, transactional sex, and forced sex. Sex can occur at any time of the day, but typically occurs in the early evening or night. Street-connected children and youth responded with various locations that sex can occur depending on the nature of the sexual encounter. Transactional sex with someone not from the street will often occur in a house or hotel. Encounters between street youth will occur in various locations including houses, verandahs, public toilets, and bushes, while rape typically occurs in the bushes and forested areas.

#### Pleasure

Children and youth connected to the streets engage in sex for pleasure and exploration, similar to other more typical adolescents. In these cases the process of seduction is similar to non street-connected adolescents courting:

“*laughing at each other, buying her new things, winking at her, walking together, they laugh at each other*” (Boy, FGD, 13–14)…“*You can hear a guy telling a girl that he dreamt of her the previous night. There is a way he wants to seduce her. So they will talk for a while and then they know each other and that is when they start dating”* (Boy, FGD, 14–17).

However, this type of courtship was rarely described in comparison to transactional relationships. Yet, both girls and boys describe the overwhelming desire to engage in sex with each other:

*“But you can also feel like doing it. If I feel like doing it even now I just look for a boy and tell him I feel like doing it and we do it, you know you cannot control those feelings when they come”* (FGD, Female, 14–17).

*“You can lust over a girl, and she may also want you. She may be infected but you won’t know, because at that time you won’t be thinking of any disease but later you will realize you have done something wrong”* (Male, 19).

#### Procreation

Having children was discussed by both street girls and boys as a reason for having sex. Participants alluded that sometimes people want a family or children to ensure they have people they leave behind when they pass away, as one participant stated: “*When you want a boy so that when you die at least you have left a child*.” (FGD, Male, 14–17).

#### Transactional sex

Street-connected children and youth frequently referred to exchanging money, food, drugs, alcohol, and gifts for sex as a primary reason for engaging in sexual acts. Boys describe seducing and luring girls using food, money, drugs, and alcohol to establish a relationship or agreement that results in sexual relations between the two parties. Transactional sex among girls was described as a regular activity and as a requirement for girls on the street to facilitate survival. As explained by two girls:

*“If your man is around, you will just be with him but if he is not around, for example if he is jailed, you cannot stay hungry as you wait for him. You will look for another man to have sex with so that you can get money. So during such times, you will be sleeping with any man who comes across as long as he has money to give you”* (Female, 21).

*“Yes. For example if I have a boyfriend who doesn’t give me money, I will prefer to go to the one with money. So I will have sex with the one with money so that he gives me money then in the evening I go back to my boyfriend. My boyfriend doesn’t need to ask me where I got the money because if he doesn’t give me money and only provides is food and clothing, then what does he expect me to do”* (Female, 15).

There are very few options for girls to earn an income on the streets, with a very limited education and skill set they are forced to engage in transactional sex. Two boys reported that: “*They don’t know how to work. They must have sex to get money*”.....“*Yes if a person doesn’t have food or money she will have to do sex to get what she wants*” (Males, 14 years). The perception among some boys is that women engage in transactional sex solely as a desire for money and not for survival due to their circumstances on the street, as one boy explained:

*“You know women love money and there are others even if you buy them glue of 20 shillings* (~0.23 USD) *or give them 50* (~ 0.57 USD) *shillings they agree to have sex. And there are others who don’t sniff glue and so you will just buy them food then they agree although they will want to do sex in the house and not in the bush”* (Male, 15 years).

Drugs and alcohol are commonly traded for sex between street-connected boys and girls, “*For me when I do it, I usually pay like 150, 200 or 100 shillings* (~ 1.14 – 2.28 USD) *although there are other girls who just need glue or alcohol. You can buy her alcohol then when she drinks, she accepts to have sex with you*” (Male, 18, Religious Barracks leader). In other cases drugs or alcohol are used to seduce a girl or lower her inhibitions, she is then usually forced to have sex in these cases to pay for the drugs and/or alcohol consumed as explained by two girls:

*“Or this glue we use… This is what confuses us mostly… You find someone buys for you glue worth twenty shillings. And he tells you that you follow him because he has used his money on you, you must pay. So you just have to sleep with him” *(Female, 19).

*“Because of so many problems like food, clothes and where to sleep. A boy will buy a girl some food then they put in some drugs that will make the girl to follow them even in the bush and then they rape you”* (Female, 15).

Boys may also participate in transactional sex with both men and women from outside the street community; however this appears to be less common than among girls. When a boy is approached for anal sex the price paid by the person requesting sex can be much more than girls receive for vaginal intercourse. Females may receive as little as 10 to 50 KES (~ 0.08-0.60 USD) as reported in interviews, but typically around 150 KES (~ 1.72 USD). This woman explained boys engaging in anal sex receive much more:

*“Yes there are men who come for boys. We call that “boot.” They will tell the boy “utanipatia boot nikulipe?” it means that, “will you do anal sex with me then I pay you?”….If it is the “boot,” they will pay around 1000 shillings* (~ 11.4 USD) *but if it is a woman they pay 500,200,150* (~ 1.72 *– 5.70 USD), or even 100 shillings”* (Female 21 years).

Additionally, it is not uncommon for adult women to seek out sexual encounters with street boys:

*“There are those women who buy boys for sex. We have a few who come from the market, they choose a boy to go and serve them then they pay for the service… I don’t know but I usually hear it is a lot of money…”* (Female, 20).

#### Sexual violence, forced and coerced sex

Boys openly discussed frequently raping and coercing girls to have sex with them on the streets. Girls are treated as sexual objects among the boys and appear to have no choice in whether they wish have sex with street-based boys. As many boys recounted: “*If a girl is approached and she refuses that is when she will be raped*”. The discussion surrounding forcing girls to have sex was so pervasive it appears that rape and coerced sex is normalized among boys and almost considered their “right” to have sex with any of the girls they wish. Extreme violence and gang rape also occur as two males described using violence and coming armed to the streets to hunt for girls:

*“…you can even threaten her with a knife or ‘kumpigangeo’….(interviewer: what is ‘kumpigangeo’?)…catching her on the throat with your elbow…ooh (Interviewer) ‘kumpigangeta’?…(Respondent) …you can be like two boys. He holds her while you rape her and after that you exchange positions…(interviewer: okay, who mostly rape girls? The big boys or the small boys?)…The big boys…big in age or size?…people around my age…” *(Male, 20 years).

*“It usually the older boys who walk in town at night. These boys you cannot see them on the streets during the day. They just come at night while armed and walking in a group of 5 or 6 then when they see a girl they rape her as a group”* (Male, 24 years).

Older men, as one boy recounts also rape young boys: “*At night. Sometimes when you are asleep someone comes behind you and sodomizes you*” (Male, 14). Street boys also discussed how market women lure them into sex with food and money and in some cases threats as described below (street boys accused of stealing are often fatal victims of mob violence if caught):

*“But if you meet her in a place like ‘Pioneer’ at the riverside, she grabs you by force. If you say you will scream, she says she will tell you are stealing from her. She says you are stealing from her”* (FGD, Male, 14–17).

#### Multiple concurrent partnerships

Multiple concurrent partnerships are frequently observed on the streets due to the transactional nature of many of the sexual partnerships. As both female and male street-connected children and youth described, they have different partners who fulfill their different needs for survival on the streets:

*“They have many because of different problems and different reasons”* (Male, 19).

*“For me, even while am with this one, I have a boyfriend and I have one who buys me food and other stuff”* (Female, 19).

Many respondents indicated they had 3 or more lovers on the streets, “*In a day you can have sex with 3 or 4 men*” (Barracks leader, Female, 20). There is a lack of monogamous commitment in partnerships, and even when a girl or a boy has a “boyfriend” or “girlfriend” they seek out additional partners to meet their other needs.

*“Very many that you can just be changing like clothes. You can have one who just belongs to you but you also have others who just have sex with you and they pay you then they go” (Female, 20).*

#### Outcomes of sexual relationships

Children and youth connected to the streets reported that heterosexual relationships could lead to both positive and negative outcomes. These relationships can provide security from violence and rape on the streets, “*but it will come a time when you will have one lover who will protect you from others. You become his property*” (Female, FGD, 14–17). Relationships may also provide basic material needs for many on the streets as one girl explained the benefits of marriage:

*“Yes there are marriages. They can get married then find a place that they will stay but they just go there at night to sleep and they spend the day at the base. Everybody is independent during the day but at night they go back together where the husband has rented a house”* (Female, 21).

Yet due to the nature of many of these relationships, including the multiple concurrent partnerships, and transactional sex, street-connected children and youth discussed many negative outcomes. Girls discussed being abandoned when becoming pregnant: “*one can make you pregnant but claim he is not responsible for it…if a man doesn’t accept responsibility you just terminate it…I terminated mine*” (Female, FDG, 14–17). While both boys and girls discussed the risk of contracting sexually transmitted diseases, including HIV/AIDS:

*“A boy can come and tell the girl that she is the one who infected him with the disease and he starts beating up that girl and maybe it is not that girl who infected him because he has slept with many girls. This girl may be accused when she doesn’t have the disease yet he contracted from a different girl” *(Male, 13).

*“It is bad because the girl may accept the man alone but then the man comes with others as a group and you know the street boys usually don’t have time to wear condoms so when they fuck you as a group, they might even tear your private parts and leave you with some diseases like gonorrhea. There is a friend of mine whom they did that and she had to be hospitalized at MTRH”* (Female, 15).

For girls, physical and sexual violence sustained in relationships can be horrific as one girl recounted the story of her fellow street girl:

*“She was married with one child. So one day she went to another street boy and unfortunately the husband came back and found her very drunk and naked with the boy sleeping under a vegetable stand (kibanda). The husband just saw the shoes and realized that it was his wife’s shoes. So when he looked keenly he found it was his friend having sex with his wife, he got hold of the girl and beat her so bad then took a bottle and inserted it in her vagina. Then he also inserted it behind (anus) and called other boys to come and sodomise her. The girl was almost dying. She is called ‘L’. So we brought ‘L’ here at MTRH and she was hospitalized then got well. She also went back to the same husband and she was still beaten, her teeth were removed and then the husband went and threw her near some water that usually swallows people called Mugoya where he had sex with her then he almost inserted a bottle in her anus but he found a certain metal and used it instead of the bottle. She got so sick and was hospitalized again but now she is well. If she goes back there again it is her own problem because we usually tell her and she doesn’t take our advice”.*

Similarly, boys who are forced into having anal sex succumb to appalling injuries as one boy offered:

*“When someone does that to you, he may damage your behinds and you may end up farting or passing feces uncontrollably… ehe…you bleed… he says you bleed…he splits your buttocks…(Others laugh)… he may sodomise him until his buttocks become red…”* (Male, FGD, 11–13).

## Discussion

Our findings provide insight into street-connected children and youth’s sexual practices, many of which not only put them at high-risk of contracting HIV and other negative health outcomes (physical and mental) but also violate numerous of their human rights as children and youth [14]. These young people urgently require protection from unlawful sexual activity, sexual exploitation, sexual abuse, maltreatment, and neglect. Kenya has recently implemented a national adolescent sexual and reproductive health policy [15]. However, to date there have been challenges in implementing and scaling up adolescent specific programmes [16], which fail to address the unique needs and concerns of street-connected children and youth. In Kenya, sexual and reproductive healthcare for young people is provided through youth-focused centres or integrated into existing services [17]. Healthcare providers face difficulties in relation to providing appropriate adolescent care in general [18]. Adolescents in both urban and rural settings report challenges with accessing care, (clinic hours, staffing, number of centres and location), educational resources, gender-focused care, and the attitude of healthcare providers towards young people in general [17]. Our findings provide information on the context and culture on the street that impacts street-connected children and youths’ sexual behaviours. This information is important for community-based organizations, healthcare providers and policy makers designing programs for adolescent reproductive health in Kenya. It highlights the need for specialized programs aimed at out-of-school youth, social protection to reduce transactional sex, and interventions addressing sexual and gender-based violence. Disseminating this evidence to community-based organizations, healthcare providers and policymakers in the region is an important step in finding strategies to safeguard the children and youth in this situation.

The commencement of children and youth’s street-involvement was described by participants as being closely linked with their sexual debut and inability to remain celibate due to peer and other pressures. This finding is not surprising given the description of street-connected children and youth engaging in transactional sex for survival once street-involved, particularly among girls, in this study and in other resource-constrained settings [3, 4, 19–21], and street initiation rites which often involve violent sexual acts among both boys and girls [8, 22]. Transactional survival sex on the streets and multiple concurrent partnerships described by street-connected children and youth put these vulnerable children and youth at very high-risk for HIV and other sexually transmitted infections. Vulnerable adolescents and those subject to poverty and abuse may be more likely to engage in transactional sex [23, 24] and the association between transactional sex and multiple concurrent partnerships and HIV-risk in sub-Saharan Africa has been well documented [25–27]. Local organizations implementing or developing prevention programs with this population should consider poverty-reduction strategies that may prevent children and youth connected to the streets from engaging in risky sexual relationships. HIV prevention programs utilizing conditional economic incentives, particularly for young girls in Tanzania and Malawi, have reduced the engagement in transactional sex and unsafe sex practices resulting in a reduction in STIs [28, 29]. In order to mitigate the harms associated risky sexual behaviour on the streets, prevention programs need to address structural as well as cultural factors of street life.

Sexual and gender-based violence (SGBV) were entrenched in girls’ discussions of sexual relations on the streets, similar to other street-connected girls in Africa [9, 19]. In Kenya, 45 % of women aged 15–49 have experienced either physical or sexual violence according to the Kenya Demographic and Health Survey and statistics from MTRH Centre for Assault Recovery of Eldoret found that 70 % of the survivors of sexual violence were below 18 years of age [30], indicating that young women in this region of Kenya are particularly vulnerable to SGBV. The outcomes of SGBV include physical, psychological, and sexual and reproductive sequelae, including HIV infection [31]. There is a need to develop programming at youth-focused centres or organizations serving street-connected children and youth that have a clear focus on reducing SGBV and disrupting this cycle of violence that is deeply embedded in street culture [8].

These findings, including sexual relations below the age of consent (16 years in Kenya), the SGBV endured by girls, forced anal sex among boys, and the exploitive survival sex, are a clear violation of the Convention on the Rights of the Child (Article 19 and 34) [14]. Sexual acts at such a young age cannot be considered consensual; in Kenya the age of consent is 16 and even if the acts occur between two minors it may be illegal, and therefore some cases may be sexual abuse, including non-consensual intercourse, touching, and exposing private parts to street-based peers, older youth, or adults. This problem is not limited to children and youth connected to the streets in Kenya alone; young age of sexual debut (< 16 years) has been reported among street-connected children and youth in other resource-constrained settings [32–34], as well as coerced and involuntarily sexual relations [4, 10, 35], and sexual abuse [9, 36]. There is a clear need for child protection agencies and governments nationally and internationally to address this tremendous violation of children’s rights and ameliorate their programs and policy to prevent these abuses [37]. While community-based organizations serving SSCY can augment their services to include sexual health education and risk-reduction programs as well as establishing shelters for girls and boys when no alternative to the street is available. Medical institutions should consider specialized sexual and reproductive health services for street-connected children and youth and other vulnerable and marginalized children and adolescents.

We recognize that this study has strengths and limitations. Strengths include that this is one of the only studies to document in detail street-connected children and youth’s perspectives and practices in relation to sexual behaviour. Secondly, this study gave a voice to some of the most marginalized children and youth allowing them to share their stories. Lastly, our study was able to recruit both boys and girls and children *on* and *of* the street to give a diversity of perspectives.

This study may also have limitations. The non-random recruitment and sampling of participants through active outreach is prone to selection bias. Children and youth who self-selected to participate in the study may be different than their peers who did not. To limit the effects of selection bias, outreach and study sensitization was performed in all areas of Eldoret where street-connected children and youth are found in an attempt to obtain a representative sample generalizable to the population. Moreover, children who agreed to participate may have been systematically different than their peers who declined. Furthermore, social desirability bias could have affected responses to sensitive questions about sexual practices and attitudes. Children may have responded to questions about sex with answers that they believed the interviewer or moderator wanted to hear. However, we believe that this was minimized due to the strong rapport and relationship the research team had with the participants. Lastly, we were unable to recruit younger girls aged 11–13 to participate in the study and therefore the experiences of older girls on the streets may not be applicable or representative of this younger group and should be interpreted with caution.

## Conclusion

Children and youth connected to the street are extremely vulnerable to sexual exploitation, violence and abuse. These findings demonstrate the need to create specialized sexual and reproductive health services and interventions for children and youth connected to the streets and evaluate their effectiveness. There is an urgent need to implement evidenced-based policy and programs to promote safer sex, reduce the harms associated with street-connected children and youth’s risky sexual behaviours, and to stop the violations and abuses sustained by children and youth connected to the streets in order to safe guard their human rights.
